# What do we know about the seed microbiome?

**DOI:** 10.1186/s40168-026-02390-0

**Published:** 2026-04-04

**Authors:** Xiangning Qi, Decai Jin, Expedito Olimi, Xiaoyulong Chen, Tomislav Cernava

**Affiliations:** 1 School of Biological Sciences, Faculty of Environmental and Life Sciences, University of Southampton, Southampton, SO17 1BJ UK; 2 Key Laboratory of Environmental Biotechnology, Research Center for Eco-Environmental Sciences, Chinese Academy of Sciences, Beijing, 100085 China; 3 Institute of Environmental Biotechnology, Graz University of Technology, Graz, 8010 Austria; 4 College of Life Sciences, Guizhou University, Guiyang, 550025 China; 5 State Key Laboratory of Green Pesticides, Center for Research and Development of Fine Chemicals, Guizhou University, Guiyang, 550025 China

**Keywords:** Plant microbiome, Seed microbiota, Plant endophytes, Seed endophytes, Plant–microbe interactions, Transgenerational inheritance

## Abstract

The seed microbiome supports plant health and increases resilience under adverse environmental conditions. Seeds are also an important vector for transgenerational transfer of the plant microbiota. Even though research over the last decade has provided valuable insights into the functional roles of seed-associated microbes, these important members of the plant microbiome remain underexplored. This review systematically highlights recently discovered key functions of the seed microbiota. It covers taxonomic composition and diversity across plant species, transmission mechanisms, functional roles in germination and seedling establishment, growth promotion, and stress resistance. The review also addresses methodological challenges and highlights critical open questions regarding assembly, spatial compartmentalization, and translation into applications. Further research into seed microbiomes has the potential to not only increase the sustainability in plant production but also to increase food security in a changing climate. Reaching such outcomes will be facilitated by mechanistic studies that will disclose the remaining secrets of plant–microbe interplay at the very first developmental stage of most plants that nowadays inhabit Earth.

Video Abstract

**Supplementary Information:**

The online version contains supplementary material available at 10.1186/s40168-026-02390-0.

## Introduction

The plant microbiome plays a crucial role in promoting plant growth and providing protection against abiotic and biotic stress [1]. It can increase resilience against environmental stresses such as salinity, drought, and cold, enhance resistance to pathogens, and also affect root structure and regulate nutrient balance [2–4]. Previous research has shown that the plant microbiome can also promote plant growth and development by modulating hormonal signalling, nutrient acquisition, and metabolism in general [5–7]. Therefore, the plant microbiome not only provides the means to improve plant health and food security but also promotes the development of sustainable agricultural practices.

**Fig. 1:**
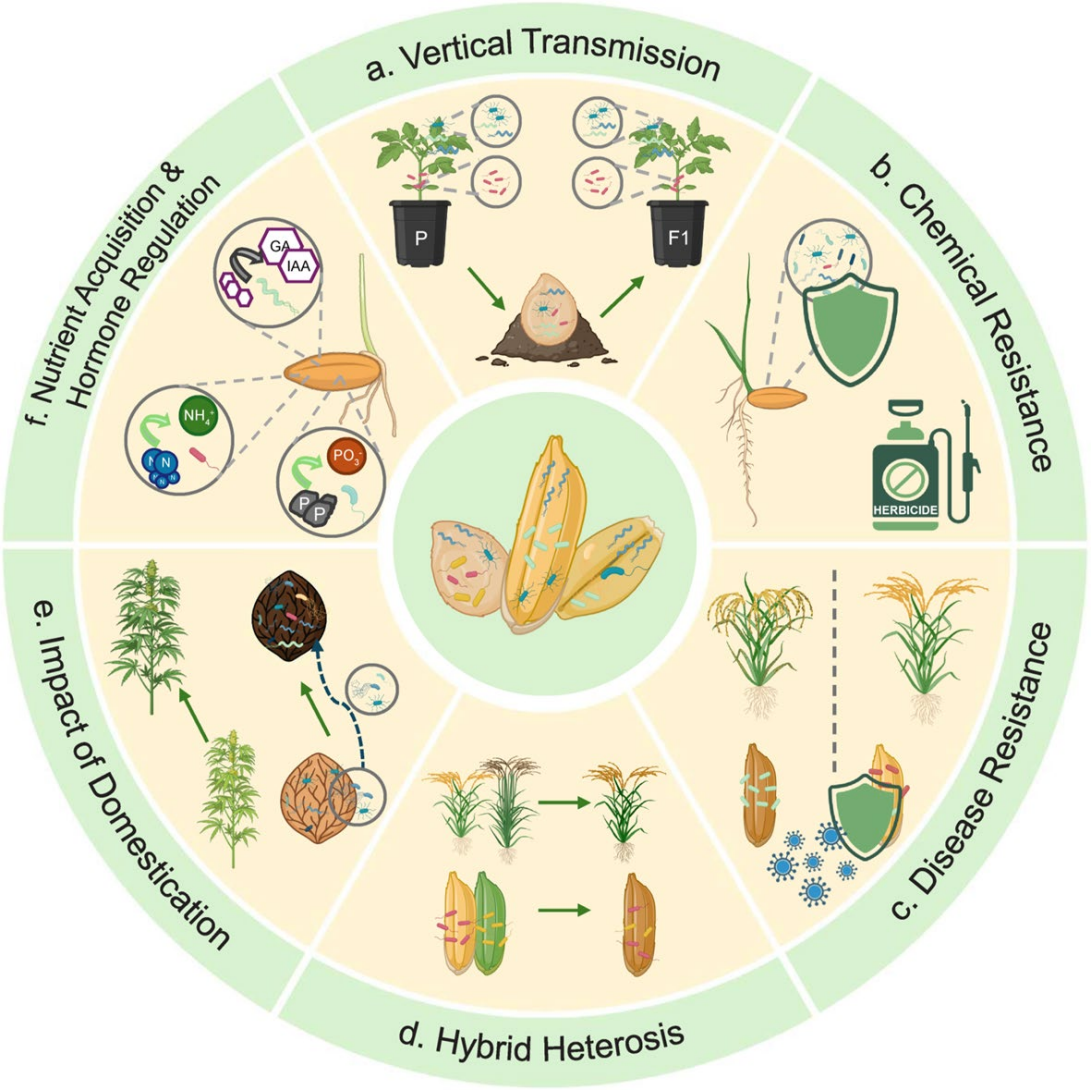
Key roles and important characteristics of the seed microbiome. **a** Vertical transmission of the seed microbiome ensures microbial inheritance between plant generations. **b** The seed microbiome can confer chemical resistance to plants, increasing their tolerance to herbicides and other pesticides. **c** The seed microbiome can enhance disease resistance by suppressing pathogens in seeds and soil. **d** Hybrid heterosis can be reflected in the seed microbiome, enhancing germination and vigor of hybrid varieties. **e** Plant domestication was shown to alter the seed microbiome, often reducing its diversity affecting key beneficial microbes. **f** The seed microbiome can promote plant growth and fitness by supporting nutrient acquisition (e.g., nitrogen fixation and phosphate solubilization) and regulating the plant’s hormone balance

Research of the last years has shown that the seed microbiome is closely associated with plant health [8]. As the earliest microbial component of plants, it is often compositionally rich and functionally diverse [9]. It also commonly consists of a fairly stable core microbiome and a variable fraction that is responsive to the environment [10]. Recent research has elucidated the seed microbiome’s critical roles in the plant life cycle and sustainable agriculture, significantly advancing our understanding of plant–microbe interactions (Fig. 1). For instance, members of the seed microbiota can promote seed germination and seedling development by synthesizing plant hormones such as auxins and enhancing nitrogen availability [11]. Furthermore, seed endophytes such as *Paenibacillus* and *Sphingomonas* have been demonstrated to protect their host plants against fungal and bacterial pathogens [12, 13]. Beyond these direct plant-beneficial effects, the seed microbiome also contributes to shaping the structure and function of soil microbial communities, thereby supporting soil health and ecosystem stability [5]. Therefore, the delivery of microbial solutions in agricultural systems will likely increasingly rely on seeds as conduits for various bioproducts [14, 15]. This is particularly due to their potential to reduce reliance on chemical pesticides, develop novel biocontrol strategies, and, consequently, promote sustainable agriculture [14, 16].

Although the role of the seed microbiome is critical, studies began to focus on it relatively late compared to microbial communities in other plant compartments [5]. In the early twentieth century, Lorenz Hiltner first recognized the role of plant-associated microbes in supporting plant health and growth, though technological limitations hindered progress at the time [14, 17, 18]. By the mid-twentieth century, the emergence of seed surface-sterilization techniques revealed the presence of microorganisms within seeds, sparking growing interest in this area [16, 19]. Early research in this field was primarily focused on seed pathology, emphasizing on the detection and management of seed-borne pathogens [20, 21]. Although fundamental research on pathogenic microorganisms remains crucial, this review focuses specifically on recent advances that have shifted the research spotlight toward the complex microbial communities associated with seeds, the vast majority of which are non-pathogenic. It should also be noted that pathogenicity can be a dynamic process in which environmental cues can fundamentally alter plant–microbe interactions [22]. Therefore, the role of certain endophytes may change during the host’s life cycle or under certain environmental conditions. The early twenty-first century marked a significant advancement in the development of high-throughput sequencing technologies, which accelerated microbiome research by enabling the rapid analysis of microbial communities, including examining the roles of the seed microbiota in plant growth, biotic stress responses, and resilience [5]. For example, metagenomics can provide comprehensive insights into the structure and genetic functionality of the seed microbiome [5, 23]; transcriptomics approaches allow us to reveal microbial gene expression patterns (in space and time), shedding light on metabolic activities, while proteomics and metabolomics have deepened our understanding of microbial functions and metabolic pathways [5, 23]. Through these approaches, researchers have gained substantial knowledge about the composition, functionality, and plant–microbe interactions related to the seed microbiome [24]. Despite these technological advances, the vast majority of research to date has focused primarily on bacterial and fungal communities within the seed microbiome. This phenomenon stems largely from the widespread use of 16S rRNA and ITS amplicon sequencing technologies, which are optimized for these communities, while historical research has also tended to focus on cultivable or easily detectable taxa [9, 10]. Consequently, other seed-transmitted microorganisms, such as viruses, oomycetes, and protozoa, remain largely unexplored despite their potential ecological significance in regulating seed viability, germination dynamics, pathogen suppression, and even horizontal gene transfer [3]. Bridging this taxonomic gap represents a critical priority for future research to achieve a more comprehensive understanding of the seed microbiome.

While the seed microbiome has gained attention during recent years, our understanding of its transmission mechanisms, ecological roles, and the influencing factors remains incomplete, with many aspects yet to be fully explored [23]. Among the key technical and conceptual challenges are the very low microbial biomass in seeds (often resulting in few or no detectable taxa per individual seed) combined with host DNA contamination [25–29], the reliance on batch-level pooling that masks inter-seed heterogeneity [26, 29], taxonomic biases limiting coverage beyond bacteria and fungi [29], and the difficulty in distinguishing vertically transmitted core taxa from transient or environmental microbes [30, 31]. These issues complicate accurate quantification, attribution of microbial origins, and causal inference of functional roles, highlighting the need for refined high-sensitivity methods and multi-scale approaches in future studies. Current research continues to face challenges, including technological limitations, difficulties in microbial cultivation, and the complexity of integrating multiple -omics techniques for comprehensive analysis of the seed microbiome [3]. Moreover, there is limited representation of seed microbiome datasets originating from agriculturally important plant/crop germplasm in particular regions of the world, such as from the indigenous communities in Africa, Oceania, and the Americas [32]. This review systematically highlights recent key findings related to seed microbiome composition, diversity, functions, transmission mechanisms, and their potential applications in agriculture. Additionally, limitations and challenges in resolving microbial entry routes into seeds, adaptation of seed endophytes to host dormancy, host-based selection mechanisms for core taxa, spatial distribution of microbes within seeds, quantification of viable microbes, and strategies for microbiome engineering are briefly discussed.

## Composition and diversity of the seed microbiome

### Core and transient microbiome members

The seed microbiome comprises a core and a transient microbiome, both exhibiting varying degrees of diversity and variability [10, 33]. The core microbiome typically comprises microorganisms shared across different plant species and remains stable under diverse environmental conditions [23, 34, 35]. Common examples include bacterial genera such as *Pantoea*, *Paenibacillus*, *Methylobacterium*, *Serratia*, *Sphingomonas*, *Pseudomonas*, and *Bacillus*; as well as fungal genera like *Cladosporium*, *Trichoderma*, and *Penicillium* [23, 36–39]. These core microbes are likely involved in mutualistic relationships with their host [40]. In contrast, the transient microbiome differs notably in composition across plant species, typically displaying low abundance but high diversity [10]. This variability may reflect the contrasting ecological conditions that surround the host plant during growth and seed production. Differences likely highlight the influence of abiotic and biotic fluctuations in plant microenvironments affecting the seed [41]. Studies suggest that bacterial communities in seeds exhibit greater uniformity, whereas fungal communities often demonstrate higher diversity [10]. The diversity of archaeal communities in seeds is generally low with a genotype unspecific compositional structure [32, 42].

### Diversity across plant species

The composition of the seed microbiome is shaped by multiple factors, such as host genotype [10], microbial transmission routes and mode of reproduction (or propagation) [24], environmental conditions [43, 44], as well as agricultural practices and postharvest seed management [43, 44], which results in significant differences in bacterial and fungal assembly across plant species. For instance, an extensive meta-analysis showed that seed microbiome composition varies across plant families [10]. Seeds of plants in the family Brassicaceae were enriched with fungi (e.g., *Dothideomycetes*), whereas Fabaceae harbored bacterial communities that varied in composition [10]. Research also indicates that seeds of Poaceae plants exhibit a high bacterial diversity, particularly in nitrogen-fixing and rhizosphere-associated taxa, which might relate to their ecological functions [10]. Generational change may also alter bacterial and fungal communities, with variations of up to 15% [45]; however, seeds of some plants in the family Leguminosae show stable multigenerational transmission [46].

Seed microbiome composition is intricately influenced by host genotype and shaped by factors such as plant lineage, domestication, and evolutionary relatedness with the associated microbial community. Seed microbial communities of rice (*Oryza sativa*) [47], cannabis (*Cannabis sativa*) [48], and alpine plants were all shown to exhibit genotype-specific microbiome differences [3], highlighting the dominant role of host selection in microbial assembly. Comparative studies across wheat cultivars (e.g., *T. aestivum* and *T. turgidum*) showed that plant cultivar significantly impacts the seed fungal composition, further emphasizing genotype-specific microbial recruitment mechanisms. Plant domestication was also shown to have influenced the seed microbiome, with highly domesticated plant cultivars showing reduced diversity, while less domesticated varieties have sometimes retained (e.g., in cannabis plants) more growth-promoting microbes, such as *Bacillus frigoritolerans* [48]. A study comparing four cereal cultivars and their respective wild relatives (e.g., *Triticum aestivum* and *Aegilops tauschii*) demonstrated that domestication resulted in a shift in microbial diversity and the structural network of seed endophytes, as well as revealed significant phylogenetic coherence between the host and its seed microbiome, providing indications for possible co-evolution [49]. In rice, differences in seed bacterial community composition correspond to the host’s genome type (e.g., GG, FF, and HHJJ), supporting the phylosymbiosis concept to explain deterministic processes in microbial assembly [50].

Seed microbiome assembly is strongly influenced by microbial transmission pathways, notably vertical transmission (parent-progeny inheritance) and horizontal transmission (acquisition from the surrounding environment). The vertical transmission pathway is central to seed microbiome assembly, including vascular, floral, and external pathways [7, 24, 39]. It ensures continuity of beneficial microbes across generations, particularly the cross-generational transfer of core endophytes that can promote plant growth and stress resistance. These microbes are preserved through reproductive tissues such as vascular bundles and floral organs, typically enhancing seedling vigor and stress tolerance. In wheat, compartment and generational analyses revealed that core endophytes are vertically transmitted [39]. They persist across successive generations, exhibiting high genomic consistency between parental and offspring seeds [46]. Moreover, temporal tracking of seed development reveals a marked increase in vertically transmitted microorganisms during late seed maturation, highlighting the dynamics in this process [46]. For instance, a large-scale study in rice demonstrated that a core set of bacterial endophytes is vertically transmitted across generations, maintaining high genomic identity between parent and offspring seeds, thus indicating stable transfer of key microbial taxa [51]. Another study analysing rice across two seasons confirmed vertical transmission of both bacterial and fungal communities from seed to seedling, as well as identified parental seeds and stem endosphere as primary sources for the subsequent seed microbiota [24, 52]. Moreover, research using source tracking revealed predominance of leaf- and root mycobiota vertical transmission in seedling, while the root and rhizosphere fungal communities of mature plants are mainly shaped by horizontal transmission from bulk soil; this highlights the importance of developmental stage- and habitat-dependent microbe transmission dynamics [53].

Besides biological factors, environmental conditions such as soil type, climate (e.g., temperature and humidity), and geographic location significantly influence seed microbiome diversity [41, 54, 55]. Studies on fonio seeds (*Digitaria exilis*) showed correlations between seed microbial diversity, local soils, and geo-climatic conditions, including pH, bulk density, and carbon content [54]. In another study, the plant microhabitat, rather than plant variety or geographic location, was shown to be the primary driver of rice seed microbiome assembly [51]. Additional factors, including agricultural practices, plant life cycle, and fruit morphology, also contribute to variations in microbial community composition and diversity [41]. Despite marked differences in microbial composition among seeds of various plant species, specific core taxa are ubiquitous across multiple hosts, forming the backbone of the seed microbiome. For instance, in *Nicotiana tabacum* (tobacco) seeds, Enterobacteriaceae dominate the core microbiome, and Lactobacilli also recurrently appear as prevalent components [56]. Moreover, meta-analyses encompassing a wide range of plant species reveal a stable core microbiota, indicating that specific bacterial and fungal taxa are conserved across seeds of different plant species [10].

## Functions of the seed microbiome

### Effects on seed germination and early seedling development

The seed microbiome significantly influences seed germination and early seedling development. During germination, specific endophytic bacteria can secrete plant hormones to break dormancy and promote sprouting [7]. Beneficial taxa like *Pseudomonas* and *Bacillus* were shown to produce antibacterial compounds (e.g., chitinases or rhamnolipids) that suppress seed-borne pathogens (e.g., *Fusarium* spp*.*), thereby increasing germination rates [16, 57]. Bacteria with cellulose-degrading capabilities can break down seed structures, releasing nutrients to accelerate germination [57, 58]. During seedling development, microbes can enhance nutrient uptake through phosphate solubilization, nitrogen fixation, and siderophore production, degrading endosperm reserves or producing hydrolytic enzymes to accelerate metabolism [5, 58, 59]. Moreover, they can secrete auxins or induce systemic resistance (ISR) to improve seedling adaptation to stresses like drought, salinity, or pathogens [23, 24]. The seed microbiome also influences the initial microbial composition of seedlings via vertical transmission, interacting with soil microbes to further shape its structure, which changes with the host plant’s developmental stages [14, 23, 24, 60]. Some taxa specifically migrate to roots and leaves, colonizing these tissues and affecting plant growth and health [5, 16]. These early-stage mechanisms lay the foundation for subsequent growth and resilience.

### Roles in promoting plant growth

Following germination and seedling establishment, seed-associated microbes can enhance nutrient availability by mobilizing nitrogen and phosphorus [43, 57]. For example, seed-borne phosphate-solubilizing bacteria (e.g., *Burkholderia* spp.) have been shown to contribute to rhizosphere phosphorus acquisition [61], and endophytic strains can improve host nitrogen accumulation and nitrogen use efficiency [11]. Studies have demonstrated that moderate nitrogen fertilization can result in the enrichment of endophytic nitrogen-fixing bacteria in rice (*Oryza sativa*) seeds, such as *Bosea* and *Aureimonas* [62]. These bacteria not only increase the seed’s microbial diversity and richness, but were also shown to correlate with elevated starch and protein content in rice grains, potentially enhancing grain quality. Regarding hormone regulation, certain microbes can synthesie plant hormones to stimulate growth and development [6, 36, 37]. For instance, *Pantoea* is a core, vertically transmitted seed endophyte in various plants [10, 32, 48], and many strains of this genus were shown to produce auxins such as indole-3-acetic acid (IAA) that can enhance plant growth [51, 63]. Multifunctionality of seed endophytes is exemplified by *Bacillus amyloliquefaciens* RWL-1. It produces gibberellins, which were shown to outperform exogenous gibberellin application, significantly enhancing rice seedling growth while also regulating the endogenous hormone balance, while also rapidly colonizing roots to improve nutrient uptake efficiency and confer disease resistance [64]. Agricultural and post-harvest practices can further amplify these growth-promoting roles. Seed inoculation with beneficial microbes (e.g., nitrogen-fixing or phosphate-solubilizing bacteria) has been shown to improve nutrient use efficiency, promote growth and yield, and enhance disease resistance [61, 65–67]. Similarly, moderate nitrogen fertilization can increase microbial diversity and richness in seeds, enrich nitrogen-fixing taxa, and thereby support nutrient acquisition and grain quality [68]. This could inform future fertilizer regimes to favor community structure shifts toward taxa that improve host performance under resource-limited conditions.

### Roles in stress resistance

Besides promoting growth, the seed microbiome can enhance plant resistance to biotic and abiotic stresses [57]. Seed microbiome members were shown to promote disease resistance by producing extracellular polysaccharides, antimicrobial compounds, virulence inhibitors, and hydrolytic enzymes, helping suppress pathogens and maintain community stability [12, 69, 70]. They can also contribute to niche competition through nutrient and spatial exclusion of invaders [51]. In terms of abiotic stresses, seed endophytes can facilitate nutrient acquisition (e.g., nitrogen fixation and phosphate solubilization), hormone production, and antioxidant regulation to resist drought and increase other forms of stress tolerance [47, 70, 71]. Heat shock proteins can support adaptation to temperature stress and metabolic adjustments can improve salt tolerance of seed-associated microbes [54].

Specific examples include *Bacillus amyloliquefaciens* RWL-1, which can also enhance disease resistance through root colonization [55], and *Sphingomonas melonis* ZJ26, which interferes with the virulence of the seed-borne pathogen *Burkholderia plantarii* [12]. The seed microbiome can also support shaping stable microbial communities in plants through intergenerational transmission, laying a foundation for long-term stress resilience [46]. Although these functions vary across plant genotypes, often showing cultivar dependency, they often remain stable amid environmental changes and are sustained across generations [46, 72].

## Open questions related to the seed microbiome

**Fig. 2:**
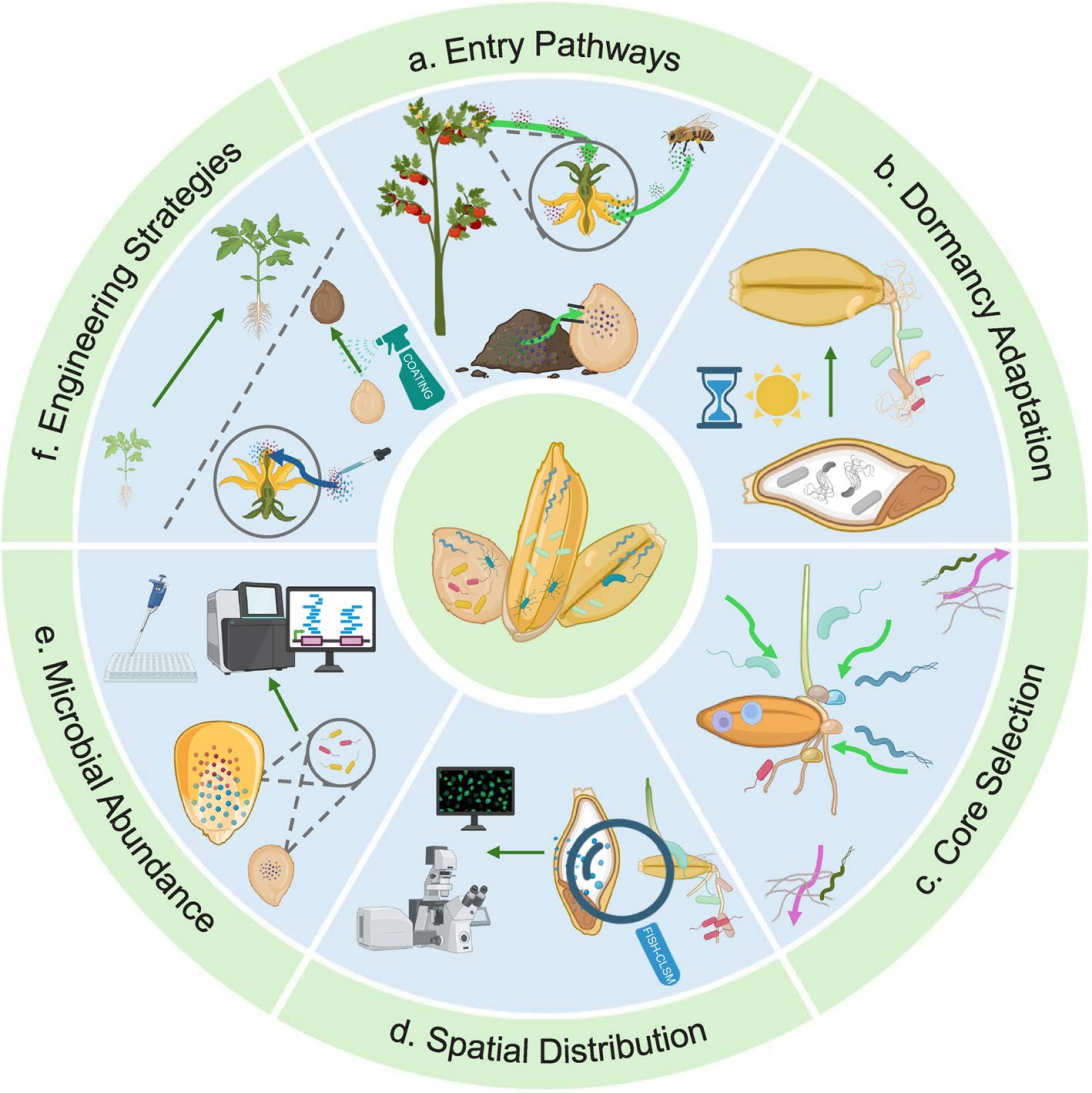
Open questions in seed microbiome research. **a** How exactly do microbes enter seeds? Is it mainly through vertical, horizontal, or combined transmission pathways? **b** How can microbes remain dormant together with the plant host, and what triggers their activation during germination? **c** How do host metabolites and immune recognition mechanisms shape the selection of core microbial taxa? **d **Why do embryos and cotyledons harbor higher microbial loads than seed coats, and how does the dominance of bacteria in roots versus fungi in shoots arise during seedling development? **e** How many viable microbes are present in individual seeds, and how does seed size influence microbial load? **f** How can seed coatings and targeted inoculation be used to optimize the introduction of beneficial microbes, and what challenges remain regarding storage and compatibility?

Despite significant progress in unravelling the mechanisms of plant–microbe interactions and the potential of seed microbiomes in sustainable agriculture, many research questions remain and need to be further explored (Fig. 2).

Understanding how microbes enter seeds is fundamental to deciphering seed microbiome assembly. Available evidence highlights two main transmission routes, which are vertical and horizontal transmission, along with selective physical barriers that shape microbial ingress [73, 74]. These barriers, including the seed coat and fruit wall, restrict microbial access and influence community composition during early colonization [71]. However, the relative contribution of vascular, floral, surface, and epiphytic transmission routes likely differs among host species, seed tissue architectures and ecological contexts, yet these are not well resolved [24]. There are some key questions, for example, to what extent do microbes use vascular, floral, or surface routes to reach the embryo, and how do plants control selective gating through tissue structure and immune recognition?

Seeds expose resident endophytic microbes to prolonged abiotic stress, selecting for taxa capable of long-term persistence and rapid reactivation at germination [5]. Multiple studies suggest that seed-associated microbes often enter metabolically quiescent or viable but non-culturable states (VBNC) during seed dormancy. Longitudinal and compartment-specific analyses further indicate that vertically transmitted taxa frequently increase in relative abundance during late seed maturation, pointing to a selective enrichment of microbial lineages that tolerate desiccation [5, 41]. During imbibition and early germination, the resumption of seed metabolic activity likely promotes the proliferation of specific microbial taxa [75, 76]. Yet, how microbes remain dormant together with the seed, and what specific host-derived signals or microbial mechanisms trigger their reactivation during germination has to be resolved in the future [77]. Moreover, we still have to find out how dormant or VBNC states can be reliably detected, and which microbial genes and metabolites are associated with these transitions. Addressing these questions will likely require combining advanced imaging and cultivation-independent techniques with functional genomics and isotope labeling approaches. In addition, deep sequencing based on shotgun metagenomics allows reconstruction of metagenome-assembled genomes (MAGs), opening new possibilities to study uncultured seed endophytes. For example, a recent study reconstructed 82 genome assemblies from barley seeds, facilitating analyses of their functional repertoires [78].

Core microbiome taxa recur across hosts and environments, consistent with strong host-driven selection [10, 23, 33]. Potential selection mechanisms include chemical filters (e.g., seed coat structure, fruit chemistry, and seed metabolites) that shape microbial colonization [10, 79], immune filtering mediated by host recognition mechanisms and genotype-specific host traits [51, 80], and microbial strategies such as motility and biofilm formation that promote persistence and colonization [81, 82]. Human-mediated processes such as domestication and breeding represent another layer of host selection: although they often reduce overall microbiome richness through altered seed chemistry or agricultural practices, functionally important taxa involved in nutrient acquisition, stress tolerance, and hormone modulation are frequently retained [23, 83]. This implies that there might be an evolutionary trade-off where core microbiome members provide essential services that partially compensate for domestication-induced vulnerabilities. However, the mechanisms underlying this filtering remain poorly resolved [79]. For instance, it is unclear how host genotypes, chemical signaling, and environmental context interact to shape core seed microbiome membership across generations. Multi-omics integration across host lineages, combined with reciprocal transplant and epigenetic profiling, may help disentangle these effects [84]. Another unresolved issue is the contribution of microbial functional traits. What microbial traits and host mechanisms govern the assembly and stability of core taxa, and how can this knowledge be used to guide breeding for microbiome-resilient crops? Trait-based microbial ecology, synthetic community experiments, and microbiome-informed breeding strategies provide promising ways forward [85–87].

Within seeds, embryos and cotyledons typically harbor higher microbial loads than the seed coat or endosperm, as observed in species like oak, melon, and rice [51, 88, 89]. During radicle emergence in maize seeds, microbes were shown to accumulate close to the tip cap and cover the whole radicle [90]. Furthermore, different studies have shown that a relative enrichment of fungal taxa occurs during seedling establishment in above-ground tissues and a predominance of bacterial assemblages in roots, with beneficial genera such as *Bacillus* and *Pseudomonas* often showing tissue-specific colonization patterns [91–93]. This micro-scale partitioning likely reflects anatomical niche differentiation driven by local physiology, nutrient gradients, and host signaling [24, 75]. However, there are still some major uncertainties, such as what is the developmental and physiological basis of this compartment-specific microbial distribution, and how conserved are these patterns across plant genotypes and species? It is also unclear how abiotic stresses like drought or temperature extremes reshape these spatial structures, and to what extent high-resolution mapping approaches, such as imaging with taxon-specific probes, laser capture microdissection, or spatially resolved metagenomics, can reveal the dynamics in microbial compartmentalization. Addressing these questions will be critical to understand how the seed microbiome contributes to plant establishment and resilience under diverse environmental conditions.

The abundance of viable microorganisms per seed was shown to vary markedly with host species, genotype, tissue compartment, developmental stage, and storage regime [5]. Conventional culture-based methods commonly underestimate microbial abundance, because many taxa enter VBNC states. In contrast, approaches such as flow cytometry, optimized viability staining, and meta-omics can reveal metabolically active or activity-marked microbes in seed coats, embryos, and cotyledons that culture-dependent methods fail to detect [94, 95]. Seed size also influences microbial carrying capacity. For example, large, endospermic seeds such as maize have been shown to host relatively rich internal endophytic communities, while in some smaller-seeded species most microbial colonization may occur post-germination [96]. Furthermore, storage conditions, particularly moisture and temperature, strongly affect microbial survival and community composition [97, 98]. Despite these insights, significant uncertainties remain regarding the exact viable microbial load in individual seeds and its variability at the single-seed level representing a key technical and conceptual challenge in seed microbiome research that warrants greater emphasis. A generally low microbial biomass means many seeds yield undetectable or single-taxon results in cultures, with some seeds even appearing sterile [26, 27, 29]. Consequently, most studies rely on batch pooling to obtain sufficient DNA, a method that averages community characteristics and obscures significant differences in composition, diversity, and biomass/load between seeds, including distinctions between sterile and colonized seeds [26, 29]. Although single-seed analysis is time-consuming, labor-intensive, and susceptible to contamination and random variation [25, 26], it reveals that individual seeds are often dominated by a single or few taxa, with substantial taxonomic variability even within the same plant or batch [76, 99]. This inter-seed heterogeneity, driven by stochastic assembly processes (limited dispersal and local filtering), has important functional implications for seed germination, seedling vigor, and plant health. Consequently, batch-level approaches may bias estimates of microbial cell numbers per seed, core taxa prevalence, transmission fidelity, and dispersal dynamics. Reliable single-seed quantification requires standardized, low-input methods (e.g., single-seed sequencing, PMA-qPCR, and flow cytometry) to capture biologically meaningful variation without pooling biases. Although these methods were successfully applied with other types of plant microbiome samples, they need to be optimized for seeds. For example, blocking dead cells with PMA (propidium monoazide) to detect the viable portion of the microbiota will only work if cell integrity is maintained during extraction. In case whole seeds are exposed to PMA, suitable pre-treatments must be used to ensure sufficient penetration of the thick-walled host cells.

Targeted breeding and engineering approaches offer promising routes to enhance seed functions such as improved germination, nutrient mobilization, and stress resilience via the seed microbiome [23, 76]. Possible strategies include the identification of host-directed selection traits through genome-wide association studies (GWAS) [100] for the identification of potential breeding targets, targeted inoculation at flowering or on developing seeds, and seed coatings to deliver synthetic communities (SynComs) [101]. Recent studies have advanced some of these efforts; for instance, Arnault et al. (2024) demonstrated that inoculating common bean seeds with diversified bacterial SynComs can profoundly modify both seed and seedling microbiota, inducing transient colonization [102]. Similarly, De-Lin et al. (2024) showed that seed-borne bacterial SynComs can inhibit seed pathogenic fungi and promote plant growth, highlighting practical applications in disease suppression and performance enhancement [103]. However, major challenges remain, including maintaining inoculum viability during storage, ensuring rapid activation post-germination, overcoming ecological incompatibility or competitive exclusion, and mitigating high inter-individual variability as well as technological limitations of seed coatings [104, 105]. Despite continuous developments that might soon enable these promising strategies at larger scale, several key questions remain. For example, how can host–microbiome co-breeding be implemented to improve seeds? What innovations in seed coatings or inoculation strategies can overcome current barriers to inoculum viability, ecological compatibility, and post-germination activation? Addressing these questions will provide critical insights for developing sustainable seed microbiome management strategies, advancing agricultural production systems toward greater efficiency and resilience.

## Conclusion and perspectives

The seed microbiome occupies a unique niche characterized by low microbial biomass, spatial isolation, and generational stability, distinguishing it from other plant compartments. Optimizing the composition of seed endophytes through microbiome engineering holds significant implications for sustainable agriculture.

Recent research advances on the seed microbiome have uncovered its taxonomic composition and diversity across different plant species, its transmission mechanisms, and its multifunctional roles in seed germination, seedling establishment, growth promotion, and stress resistance. As a dynamic, vertically transmitted microbial community, the seed microbiome not only supports early plant development but also contributes to long-term plant adaptation through nutrient mobilization, hormone regulation, pathogen suppression, and intergenerational stability.

Despite these advances, fundamental gaps remain in our understanding of this critical compartment. Key uncertainties include the specific paths and regulatory mechanisms that allow microbes to enter and survive within seeds; the physiological and molecular basis for microbial dormancy and reactivation during germination; host and microbial traits determining core community assembly and intergenerational stability; and developmental drivers of internal compartmentalization within seeds and seedlings. Methodological challenges, such as low microbial biomass, host DNA contamination, taxonomic bias, and the need for single-seed resolution, still hinder progress in linking microbial community composition to functional outcomes.

Resolving these unanswered questions requires a comprehensive approach, such as integrating multi-omics technologies with spatially resolved techniques, leveraging large-scale datasets from various resources, and conducting synthetic community experiments to explore the underlying principles. Recent discoveries in plant microbiome research indicate that *Microbiome* genes (*M* genes) are promising breeding targets in plants that allow microbiome optimization via host genetics [106]. These genes regulate either host immunity or chemical signaling in plants to recruit specific microbial taxa. By developing plant varieties that shape their seed-endophytic communities via *M* genes, new avenues for practical applications could emerge due to the transformative potential for sustainable agriculture. In addition, targeted inoculation with beneficial seed endophytes, bio-priming, or advanced seed coatings could reduce reliance on chemical inputs, enhance resilience to climate stresses, improve nutrient use efficiency, and ultimately boost crop yields, quality, and food security. Finally, by filling the remaining knowledge gaps, future research will position the seed microbiome as a powerful tool for developing resilient, resource-efficient cropping systems in a changing climate.

## Data Availability

No datasets were generated or analysed during the current study.
